# The Effects of an Acute Strongman Competition on Electromyographic Responses of the Shoulder Girdle Complex

**DOI:** 10.3390/life16030477

**Published:** 2026-03-16

**Authors:** Rafał Studnicki, Julia Wasilewska, Igor Z. Zubrzycki, Magdalena Wiacek

**Affiliations:** 1 Department of Physiotherapy, Medical University of Gdańsk, 7 Dębinki Street, 80-211 Gdańsk, Poland; julia.wasilewska@gumed.edu.pl; 2 Department of Medical and Health Sciences, Radom University, Chrobrego 27, 26-600 Radom, Poland; igorzubrzycki@gmail.com (I.Z.Z.); m.wiacek@urad.edu.pl (M.W.)

**Keywords:** muscle injuries, rotator cuff, extreme sports, weightlifting, muscle physiology

## Abstract

Background: Strongman competitions impose extreme mechanical and metabolic stress on the shoulder girdle, yet quantitative neuromuscular responses under real competition conditions remain poorly characterized. Methods: Ten elite strongmen (Tier 4) and ten age-matched trained controls (Tier 2) completed an official Strongman Champions League competition protocol. Surface EMG was recorded from seven shoulder-girdle muscles during maximal voluntary contraction (MVC) trials performed immediately before and after competition. Normalized RMS amplitudes were expressed as a relative EMG index (% group peak) and analyzed using linear mixed-effects models with Benjamini–Hochberg false discovery rate (FDR) correction. Results: Within-group analyses revealed no generalized pre–post reductions in normalized EMG amplitude in either group after FDR correction. However, the control group demonstrated consistent negative pre–post trends with moderate-to-large effect sizes across several muscles, particularly for mean and median descriptors. In contrast, elite strongmen exhibited smaller and more variable changes without a systematic decline. Difference-in-differences analysis showed that temporal changes generally favored the elite group. After FDR adjustment, a significant interaction was identified for the median lower trapezius amplitude (ΔΔ = 33.76 ± 9.13, pFDR = 0.021), indicating relatively greater preservation of neuromuscular activation in elite strongmen compared with controls. No contrast demonstrated a greater decline in the elite group. Conclusions: Although most effects did not survive correction for multiple testing, the observed effect-size patterns and a significantly lower trapezius interaction suggest greater stability of neuromuscular activation in elite strongmen compared with trained, non-specialized controls. These findings support muscle- and metric-specific fatigue resistance associated with long-term strongman training.

## 1. Introduction

Strongman competitions demand extraordinary muscular strength, rapid adaptability, and the ability to execute dynamic, high-intensity tasks under extreme conditions. As Nimphius et al. [1] demonstrated, strength and power critically determine performance in movements requiring rapid directional changes—a hallmark of strongman events. Understanding how strength, speed, and coordination interact is, therefore, essential for defining the limits of strongman performance. Accordingly, evaluating competition-induced fatigue effects on maximal voluntary contraction (MVC) of the shoulder-girdle musculature during the Strongman Champions League can advance biomechanical and physiological models of performance in this sport.

Strongman preparation integrates strength, endurance, and speed training, eliciting multiple fatigue mechanisms that differentially affect muscular output [2]. The shoulder complex—which consists of the deltoid, rotator cuff, and scapular stabilizing muscles—plays a central role in force generation and joint stability [3]. Fatigue-induced disruptions in these synergistic muscles compromise force production and joint integrity, thereby increasing the risk of injury during high-load tasks. Specifically, Saeterbakken et al. [4] demonstrated that increasing stability demands during the chest press (dumbbells vs. barbell and Smith machine) resulted in substantially lower 1-RM loads. At the same time, electromyographic activity of the prime movers (pectoralis major and anterior deltoid) remained comparable across conditions when lifting at 1-RM intensity. In contrast, antagonist and stabilizing muscles exhibited divergent activation patterns, with greater biceps brachii activity and reduced triceps brachii activity observed under higher stability requirements, reflecting increased demands for joint stabilization rather than enhanced force production [4].

Neuromuscular fatigue encompasses both central (reduced CNS motor-unit recruitment) and peripheral (metabolic by-product accumulation, impaired excitation-contraction coupling) factors [5]. Central fatigue—driven by neurotransmitter depletion and neuromodulatory changes—limits voluntary activation even before peripheral exhaustion [6,7]. Peripheral fatigue then further suppresses the motor drive to protect against structural failure [8]. In strongman competition, central fatigue may hinder shoulder stabilizer activation across successive events, while peripheral fatigue reduces force output under sustained isometric holds. Quantifying the combined effects of these factors on MVC under competition-style loading will elucidate how fatigue undermines performance and guide targeted training competition exposures.

Surface electromyography (sEMG) provides a powerful means to assess fatigue-related changes in activation patterns during dynamic and isometric tasks [9] and allows distinguishing fatigue effects in voluntary contractions [10].

Moreover, advancements in multielectrode array technology enable the isolation and characterization of individual MU discharge patterns and their contributions to force-generating mechanisms [11,12]. Of particular relevance to athletic performance and injury prevention is the rate of force development (RFD), which has been shown to correlate strongly with both motor unit (MU) discharge rate and recruitment velocity [13,14].

The temporal fluctuations observed during steady-state isometric contractions can be accurately predicted by applying a low-pass filter to the neural drive directed to the musculature, thus elucidating a functional linkage between common motoneuronal oscillations and force-tracking precision [15]. Collectively, these insights substantiate the utility of sEMG as a versatile research modality in both controlled laboratory investigations and in situ field assessments [16].

Research comparing muscle synergy structures in experienced powerlifters and untrained individuals reveals significant inter-group variability, particularly during complex lifts such as the bench press. For instance, Kristiansen et al. found that elite powerlifters exhibited distinct muscle synergy patterns during the bench press compared to untrained individuals, indicating a developed proficiency in muscle recruitment that can lead to optimal performance outputs [17]. Conversely, greater variability in muscle activation has been observed in recreational lifters, suggesting a different level of neuromuscular adaptation and potentially less refined lifting techniques [18].

Insights from EMG studies indicate that practitioners and athletes can effectively tailor training regimens by understanding the differences in neuromuscular activation among strength athletes. Tillaar and Sæterbakken noted that experiencing fatigue can lead to variations in muscle activation, impacting overall performance during critical lifting sessions and adding another layer of complexity to training strategies [19].

It has been shown that sEMG data normalization is fundamental for meaningful comparisons across individuals and conditions. Various methods, such as using peak or mean values during tasks, significantly affect the final results and their interpretations. Burden points out that the magnitude of processed EMGs can vary with normalization method, underscoring the importance of standardized practices in EMG research [20]. Furthermore, variations in MVC measurements can introduce intersubject variability, complicating the interpretation of results from strongman events, where such fluctuations may be consistent due to the physical demands of competition.

Despite a growing body of electromyographic research in strength-trained populations such as powerlifters, Olympic weightlifters, and overhead athletes, the myoelectric characteristics of elite strongman competitors remain virtually unexplored. Existing EMG studies have primarily focused on isolated resistance exercises performed under laboratory conditions, often emphasizing prime movers rather than the coordinated activation of shoulder-girdle stabilizers under extreme, competition-specific loading. To date, no studies have systematically examined normalized surface EMG responses of multiple shoulder muscles in elite strongmen during an actual competition setting. This absence represents a critical gap in the literature, given the uniquely high external loads, complex multi-joint demands, and cumulative fatigue characteristic of strongman events.

Elite strongman competitions consist of multiple high-load events performed within a single competition day, requiring repeated maximal or near-maximal efforts separated by externally imposed rest intervals. Collectively, these events impose substantial mechanical stress and cumulative neuromuscular fatigue on the shoulder girdle, which is repeatedly required to transmit force, stabilize external loads, and maintain joint integrity under extreme conditions. In the present study, the competition was therefore not treated as a performance outcome but rather as a standardized, ecologically valid fatigue stimulus. By assessing normalized electromyographic activity during maximal voluntary contraction (MVC) trials immediately before and after the competition, it was possible to quantify acute competition-induced alterations in shoulder-girdle muscle activation. This approach provides a mechanistic framework for evaluating neuromuscular fatigue resistance and stabilization capacity under real-world competitive loading, which cannot be fully replicated using isolated laboratory exercise protocols.

Therefore, this study aimed to examine competition-induced changes in normalized electromyographic activity of the shoulder-girdle musculature during maximal voluntary contraction trials in elite strongman athletes and to compare these responses with those of recreationally active controls under matched task conditions. Moreover, the present investigation focuses on competition-induced changes in normalized sEMG amplitude and does not directly resolve motor-unit recruitment or discharge behavior.

Based on the extreme mechanical and metabolic demands imposed by strongman competition and the established neuromuscular adaptations associated with long-term exposure to such loading, the following a priori hypotheses were formulated. (1) elite strongman athletes would preserve normalized electromyographic activation of shoulder-girdle muscles during MVC trials following competition; (2) recreationally trained controls would exhibit greater reductions in normalized EMG activation following competition; and (3) competition-induced changes in neuromuscular activation would be muscle-specific, with stabilizing shoulder complex muscles—particularly the trapezius (upper and lower), serratus anterior, and infraspinatus—exhibiting different fatigue responses compared with the deltoid muscles.

## 2. Materials and Methods

### 2.1. Ethics

The study was approved by the Independent Bioethics Committee for Scientific Research at the Medical University of Gdańsk (13 September 2024, Resolution No. NKBBN 372/2024). After explaining the protocol in clear terms, written informed consent was obtained from all participants. The study adhered to the principles outlined in the Declaration of Helsinki.

### 2.2. Study Participants and Sample Allocation

Ten elite strongmen (Study Group, SG)—each with at least five international Strongman Champions League (SCL) appearances in the preceding year and classified to Tier 4: Elite/International Level [21]—were recruited at the Polish CSL competition. For both groups, eligible volunteers were healthy males aged 18 or older; exclusion criteria included lack of consent, recent cessation of resistance training, lower-limb or spine surgery, lower-limb injury or joint pain within 3 months (hip, knee, ankle), lower-limb joint hypermobility, neurological disorders, and connective-tissue diseases. Full details of potential risks and benefits of the examination process were provided, and written informed consent was obtained before testing. The control group (CG), classified as Tier 2: Trained/Developmental [21], comprised males of similar age who underwent a standardized, strongman-like protocol.

The study was conducted during an official Strongman Champions League competition. The competition was held within a single competition day and consisted of multiple high-load events performed sequentially according to the standardized SCL schedule. Rest intervals, determined by the organizers, separated each event, as is inherent to the competition format. All events were completed on the same day; no multi-day competition format was used. During the competition, participants did not receive any additional training, as athletes were engaged exclusively in the scheduled competitive events. Prior to participation, athletes were not instructed to modify or restrict their habitual training routines, reflecting standard competition practice in elite strongman events. Consequently, no formal pre-competition exercise abstention period was imposed beyond the athletes’ normal preparation routines.

Participants were allocated to the experimental (SG) or control (CG) group and performed five distinct loading tasks matched for duration but differing in absolute load magnitude and implements. Strongman athletes performed all tasks during an official competition using the standardized loads prescribed by the event organizers [22]. First, both groups completed an isometric deadlift task held for 60 s: the experimental group sustained 350 kg, whereas the control group sustained 100 kg. Second, a dynamic tossing task over a fixed distance of 350 cm was performed for 60 s: SG executed a Bag-Toss Medley using sequential implements of 18, 20, 22, 24, and 26 kg, while the CG performed a medicine-ball throw over the same 350 cm distance, as prescribed by the official competition rules, using balls weighing 14, 16, 18, 20, and 22 kg. Third, participants performed a load-carrying exercise for 60 s: SG carried a 150 kg frame, whereas CG carried an 80 kg trap bar. Finally, both groups completed a 75-s sled-pull task: SG pulled 180 kg, and CG pulled 50 kg.

### 2.3. Experimental Procedures

The study was conducted during the Strongman Champions League (CSL) competition. Before participating in the experiment, participants received a detailed oral explanation of the study procedure. Participants completed one familiarization trial before data collection; the results were not recorded. Two research team members supervised the test procedure, ensuring that the study order was followed. EMG testing was conducted twice. The first test was conducted before the competition began. The second assessment was conducted after the competition concluded. The competition consisted of the following events: Truck-Push, Frame-Carry for Time, Bag-Toss Medley, Log-Lift for Repetitions, and Power-Stairs.

**Figure 1:**
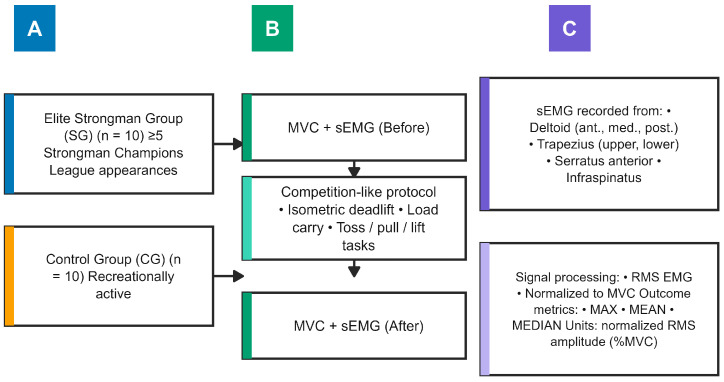
Experimental setup and study timeline. Participants were allocated to a study group (elite strongmen, Tier 4: Elite/International Level) and a control group (Tier 2: Trained/Developmental). Surface electromyography (sEMG) recordings were obtained during maximal voluntary contraction (MVC) trials before and immediately after a competition. EMG activity was recorded from seven shoulder-girdle muscles and analyzed as normalized RMS amplitude (%group peak scaled), including maximal (MAX), mean (MEAN), and median (MEDIAN) outcome metrics (**A**–**C**). Specific level of study time line: (**A**)—sample collection; (**B**)—experimental procedure; (**C**)—data analysis.

The experimental scheme is shown in Figure 1.

### 2.4. Surface EMG Assessment

Surface electromyography (sEMG) was recorded from seven muscles of the dominant (as defined by the subject) upper limb: anterior deltoid, medial deltoid, posterior deltoid, upper trapezius, lower trapezius, serratus anterior, and infraspinatus during maximal voluntary isometric contraction (MVC) trials. MVC force output was not directly quantified; therefore, EMG outcomes reflect neuromuscular activation during MVC trials rather than maximal force production per se.

Skin preparation and electrode placement were performed according to SENIAM guidelines [23], including shaving, abrasion, and alcohol cleansing. Bipolar Ag/AgCl surface electrodes (1 cm^2^ contact area; Sorimex, Toruń, Poland) were placed with an interelectrode distance of 20 mm (center-to-center) in accordance with SENIAM recommendations. Skin preparation included shaving (if necessary), light abrasion, and cleansing with 70% isopropyl alcohol to reduce impedance. Electrodes were aligned parallel to muscle fiber orientation, and a reference electrode was placed over an electrically neutral bony prominence.

Signals were acquired using a TeleMyo DTS wireless EMG system (Noraxon USA Inc., Scottsdale, AZ, USA) with the following parameters:

÷ Differential amplification gain: 300
÷ Sampling frequency: 1500 Hz
÷ Analog-to-digital resolution: 16-bit
÷ Hardware band-pass filter: 15–500 Hz

Raw signals were visually inspected to identify and exclude segments containing movement artifacts or baseline noise contamination.

All signal processing was performed using MyoResearch 2.8 software (Noraxon USA Inc.). The following processing steps were applied: (1) Raw EMG signals were digitally band-pass filtered (15–500 Hz) using a zero-phase filter. (2) Signals were full-wave rectified. (3) The rectified signal was smoothed using a root-mean-square (RMS) algorithm with a 300 ms moving window.

The stable plateau portion of each 3-s MVC trial was visually identified for analysis.

Maximal voluntary contraction (MVC) trials were performed to provide a standardized reference task for surface electromyographic (sEMG) signal acquisition. During MVC trials, participants were instructed to perform a brief maximal isometric contraction of the target muscle group against manual resistance. Only sEMG signals were recorded during MVC trials. No external force, torque, or mechanical output was measured, and no force–time curve was obtained. MVC trials were therefore used solely as a reference contraction for sEMG recording procedures and were not used to derive mechanical outcome variables.

The following amplitude-based descriptors were extracted from the RMS-processed EMG signal: maximal RMS amplitude (MAX), mean RMS amplitude (MEAN), and median RMS amplitude (MEDIAN). No frequency-domain variables (e.g., median frequency or mean power frequency) were analyzed in the present study.

### 2.5. Maximal Voluntary Isometric Contraction (MVC) Standardization and Normalization Procedure

MVC trials served as standardized isometric activation tasks rather than direct measurements of maximal force output.

To facilitate comparison of within-group changes across muscles and amplitude descriptors, sEMG amplitude outcomes were expressed as a relative EMG index using group-specific peak scaling. For each muscle × descriptor, values within a given group were divided by the maximum observed value in that group and multiplied by 100. Thus, values represent a percentage of the group’s observed peak (0–100 scale) rather than participant-level normalization to maximal voluntary contraction (MVC).

Moreover, EMG amplitude was normalized to reduce inter-individual variability caused by electrode placement, subcutaneous tissue, and signal impedance. While maximal voluntary isometric contractions are commonly used as the normalization reference, alternative methods such as peak or mean EMG within analyzed tasks have been employed in the literature when force-matched MVC references are impractical or not representative of task-specific activation [20,24,25]. In particular, normalization to the maximum amplitude within a dataset (dynamic peak normalization) provides a reproducible internal reference that preserves proportional within-dataset differences [25,26].

This scaling supports evaluation of within-group change patterns and group × time interaction effects in relative units, but it does not preserve absolute between-group differences in EMG magnitude. MVCs were executed as isometric contractions, and therefore, joint angles and muscle lengths were held constant during each trial.

Maximal voluntary isometric contraction (MVC) trials were performed using standardized manual resistance aligned with the primary anatomical function of each tested muscle. All contractions were performed with controlled body positioning to minimize compensatory movements and ensure reproducibility.

Testing of all studied muscles was performed in a seated position. Participants were seated upright without back support, with the trunk in neutral alignment and feet flat on the floor.

For the anterior deltoid, the tested shoulder was positioned at 90° of flexion in the scapular plane (30° anterior to the frontal plane) with the elbow fully extended and the forearm in neutral rotation (thumb facing medially). The examiner stabilized the ipsilateral scapula and superior shoulder girdle to prevent elevation or trunk compensation. A posteriorly directed manual resistance was applied at the distal humerus immediately proximal to the elbow joint, perpendicular to the longitudinal axis of the humerus. Participants were instructed to maintain shoulder flexion against maximal resistance without trunk movement.

For the medial deltoid, the shoulder was abducted to 90° in the frontal plane, with the elbow extended and the humerus maintained in neutral rotation. The examiner stabilized the superior shoulder girdle to prevent scapular elevation and minimized lateral trunk flexion. A downward (inferiorly directed) resistance was applied at the distal humerus just proximal to the elbow joint, perpendicular to the humeral shaft. Participants performed maximal isometric shoulder abduction.

For the posterior deltoid, the shoulder was flexed to 90°, with the elbow extended and the humerus maintained in neutral to slight external rotation. From this position, participants performed horizontal abduction in the transverse plane. The examiner stabilized the scapula and trunk to prevent thoracic rotation. An anteriorly directed manual resistance was applied at the distal humerus proximal to the elbow joint, perpendicular to the humerus. Participants resisted the applied force isometrically.

Infraspinatus testing was conducted with the shoulder adducted at the side (0° abduction) and the elbow flexed to 90°. A towel roll (approximately 2–3 cm thickness) was placed between the elbow and trunk to standardize humeral alignment and reduce compensatory deltoid activation. The forearm was maintained in neutral rotation. The examiner stabilized the elbow against the trunk and applied a medially directed resistance at the distal forearm proximal to the wrist joint, perpendicular to the forearm. Participants performed maximal isometric external rotation while maintaining humeral stabilization.

For the upper trapezius, participants performed scapular elevation (shoulder shrug) without cervical lateral flexion. The examiner applied an inferiorly directed manual resistance over the acromion, perpendicular to the direction of elevation. Participants maintained maximal isometric scapular elevation.

For the lower trapezius, the tested shoulder was elevated to approximately 135° in the scapular plane (seated Y position) with the elbow fully extended and the humerus in external rotation (thumb pointing upward). The trunk was maintained against the backrest to prevent lumbar extension or thoracic compensation. Participants were instructed to perform scapular depression and retraction while maintaining the arm position. The examiner applied a combined inferiorly and anteriorly directed manual resistance at the distal forearm proximal to the wrist joint, aligned with the scapular plane. Participants maintained maximal isometric contraction against resistance.

For the serratus anterior, the trunk was maintained in a neutral upright posture against the backrest to minimize thoracic flexion or rotation. The tested shoulder was positioned at 90° of flexion in the scapular plane (approximately 30° anterior to the frontal plane) with the elbow fully extended and the forearm in neutral rotation. From this position, participants were instructed to perform scapular protraction (“reach forward without bending the elbow”) while maintaining the shoulder elevation angle. The examiner stabilized the contralateral shoulder girdle and trunk to prevent compensatory trunk rotation, thoracic flexion, or shoulder horizontal adduction. A posteriorly directed manual resistance was applied at the distal forearm just proximal to the wrist joint, aligned with the longitudinal axis of the forearm, and opposing the direction of protraction. Participants were instructed to maintain maximal scapular protraction against resistance without elbow flexion, trunk movement, or shoulder elevation.

Because all normalization contractions were isometric, no shortening or lengthening velocity was present, and ranges of joint motion were not applicable.

Real-time visual biofeedback was provided via the EMG acquisition software, and the same investigator delivered strong standardized verbal encouragement throughout all trials to promote maximal and consistent effort. Each muscle was tested three times with 60–90 s rest between trials to minimize fatigue carryover. The highest of the three MVC trials was retained as the representative value for analysis.

All MVC trials were performed against examiner-applied manual resistance (no dynamometer or fixed rig). To standardize the ‘immovable’ condition, resistance was increased rapidly to match the participant’s effort and then held as rigidly as possible. Meanwhile, the participant was instructed to maintain the target joint position without visible movement. The same investigator applied resistance across all participants and sessions, using consistent hand placement and force direction.

### 2.6. Statistical Analysis

Data were normalized using a within-group maximum scaling procedure to express all observations as a percentage of the highest observed value within each data vector. For each set of measurements, only finite values were considered when determining the maximum. Individual values were subsequently divided by this group-specific maximum and multiplied by 100, yielding normalized values expressed as percentages of the group maximum. This approach preserves relative proportions among observations while minimizing the influence of between-group variability on absolute magnitudes, thereby facilitating meaningful comparisons across groups or experimental conditions. To ensure numerical robustness, vectors containing no finite values returned missing values, whereas cases in which the maximum was non-finite or equal to zero were assigned zero values.

Thus, MVC trials served as the physiological reference condition, while numerical normalization was implemented using within-group maximum scaling to stabilize between-group variance.

All statistical analyses were performed using R (version 4.5.2, R Foundation for Statistical Computing, Vienna, Austria). Linear and mixed-effects models were fit using the lme4 and lmerTest packages, and estimated marginal means were obtained using the emmeans package. For each region × category subset, the primary model was specified as:

$$Y_{igt}=\beta_{0}+\beta_{1}{\mathrm{Group}}_{g}+\beta_{2}{\mathrm{Time}}_{t}+\beta_{3}({\mathrm{Group}}_{g}\times {\mathrm{Time}}_{t})+\varepsilon_{igt},$$

where $Y_{igt}$ denotes the outcome variable, and $\beta_{3}$ represents the DiD estimand.

When repeated measurements were present within individuals (i.e., when the same subject contributed more than one observation within a given region × category subset), a linear mixed-effects model with a subject-specific random intercept was fit:

$$Y_{igt}=\beta_{0}+\beta_{1}{\mathrm{Group}}_{g}+\beta_{2}{\mathrm{Time}}_{t}+\beta_{3}\left({\mathrm{Group}}_{g}\times {\mathrm{Time}}_{t}\right)+b_{i}+\varepsilon_{igt},$$

where $b_{i}∼N(0,\sigma_{b}^{2})$ represents between-subject variability.

When no repeated measurements were present within subjects, a standard ordinary least squares (OLS) linear model was used instead. This conditional modeling strategy prevented overparameterization and avoided non-estimable contrasts that arise when random effects are applied to non-repeated data.

Random effects were not specified for data source identifiers when these were perfectly confounded with treatment group, as such structures preclude separation of group effects from random intercepts. Effect sizes were interpreted descriptively and were not considered evidence of meaningful change in the absence of FDR-significant contrasts.

Estimated marginal means for each group × time combination were computed using the emmeans package. The DiD effect was defined as:

$$\Delta \Delta =\left({\mathrm{Study}}_{After}-{\mathrm{Study}}_{Before}\right)-\left({\mathrm{Control}}_{After}-{\mathrm{Control}}_{Before}\right).$$

This contrast was estimated directly from the whole group × time marginal mean grid. For each region × category combination, point estimates, standard errors, degrees of freedom, test statistics, and *p*-values were extracted. Models yielding non-estimable contrasts (e.g., due to rank deficiency or zero within-cell variance) were flagged and excluded from inferential interpretation.

Given the multiple region × category comparisons, *p*-values were adjusted for multiple testing using the Benjamini–Hochberg false discovery rate (FDR) procedure. Adjusted *p*-values ≤ 0.05 were considered statistically significant.

## 3. Results

Analysis of chronological age, body height, and body mass provides the following values for SG: 32.9 ± 4.6 yrs. of age, 194.3 ± 5.5 cm, and 151.6 ± 10.4 kg, respectively, and for CG: 30.9 ± 2.5 yrs. of age, 188 ± 7.8 cm, and 108 ± 30 kg.

**Figure 2:**
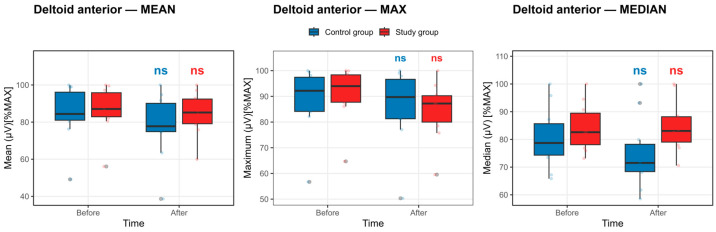
Deltoid anterior muscle activation before and after the competition exposure. Statistical significance *p* < 0.05, defined by the Benjamini–Hochberg false discovery rate, else nonsignificant. Boxplots show median and IQR; whiskers = 1.5 × IQR. ns: non-significant.

Deltoid anterior muscle: In contrast to an expected fatigue-related decline, no statistically significant within-group reductions in deltoid anterior muscle activation were observed following the competition exposure in either the study or the control group. As shown in Figure 2, the boxplots of maximal, mean, and median EMG amplitudes demonstrate substantial overlap between pre- and post-competition exposure measurements in both groups, with no consistent downward shift in central tendency. These visual findings are corroborated by Supplementary Table S1, where none of the before–after contrasts for deltoid anterior activation reached statistical significance after BH–FDR correction (all pFDR > 0.40). Although moderate-to-large standardized effect sizes were observed in the control group, the wide confidence intervals and nonsignificant adjusted *p*-values indicate high variability rather than a systematic fatigue-induced suppression of muscle activation. Collectively, these data suggest no statistically significant change in deltoid anterior neuromuscular activation across repeated maximal efforts, with no evidence of maladaptive fatigue in either group.

**Figure 3:**
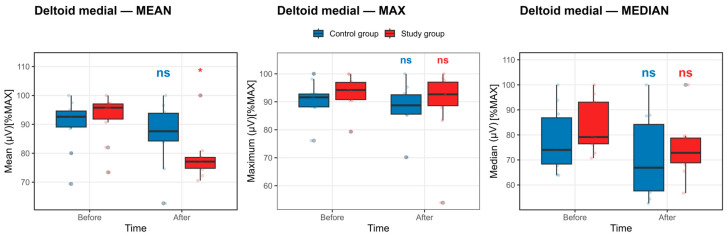
Deltoid medial muscle activation before and after the competition exposure. Statistical significance *p* < 0.05, defined by the Benjamini–Hochberg false discovery rate, is denoted by *, else nonsignificant. Boxplots show median and IQR; whiskers = 1.5 × IQR. ns: non-significant.

Deltoid medial muscle: Analysis of deltoid medial muscle activation revealed a selective within-group change that was dependent on the EMG signal descriptor. As illustrated in Figure 3, the boxplots of maximal and median EMG amplitudes showed substantial overlap between pre- and post-competition exposure measurements in both the control and study groups, with no consistent shifts in median or interquartile range. In contrast, the distribution of mean EMG amplitude in the study group showed a discernible downward displacement following the competition exposure.

These graphical observations are corroborated by the statistical outcomes reported in Supplementary Table S2. In the study group, a significant reduction was detected for the mean EMG amplitude (Δ = −13.87 ± 4.13), which remained statistically significant after Benjamini–Hochberg false discovery rate correction (pFDR = 0.026), accompanied by a large standardized effect size (Cohen’s d = −1.00, 95% CI −1.75 to −0.21). No corresponding significant changes were observed for maximal or median EMG amplitudes in the study group after FDR adjustment. Similarly, all before–after contrasts for the control group failed to reach statistical significance across maximal, mean, and median parameters (all pFDR ≥ 0.41).

Overall, the deltoid medial results indicate a parameter-specific reduction confined to the mean EMG signal in the study group, rather than a uniform decrease across all EMG descriptors. This pattern suggests nuanced modulation of deltoid medial activation rather than generalized fatigue-related suppression of neuromuscular output.

**Figure 4:**
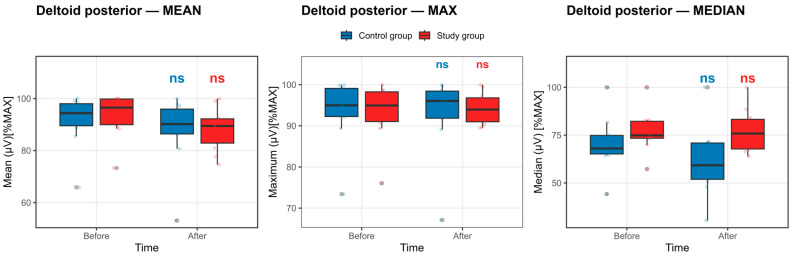
Deltoid posterior muscle activation before and after the competition exposure. Statistical significance *p* < 0.05, defined by the Benjamini–Hochberg false discovery rate, else nonsignificant. Boxplots show median and IQR; whiskers = 1.5 × IQR. ns: non-significant.

Deltoid posterior muscle: Evaluation of deltoid posterior muscle activation demonstrated stable neuromuscular output across the competition exposure period in both groups. As shown in Figure 4, the boxplots of maximal, mean, and median EMG amplitudes exhibited substantial overlap between pre- and post-competition exposure measurements, with no consistent shifts in median or interquartile range indicative of a systematic reduction in activation.

These visual findings are supported by the statistical analyses presented in Supplementary Table S3. None of the within-group before–after contrasts for deltoid posterior activation reached statistical significance following Benjamini–Hochberg false discovery rate correction in either the control or study group (all pFDR ≥ 0.41). Although moderate standardized effect sizes were observed for selected parameters—particularly for mean and median EMG amplitudes in the control group—the associated confidence intervals were wide, and all adjusted *p*-values were nonsignificant, indicating considerable inter-individual variability rather than a consistent fatigue-related effect.

Collectively, the concordance between the boxplot distributions and the mixed-effects model results indicates no statistically significant change in deltoid posterior muscle activation following repeated maximal efforts, with no evidence of parameter-specific or global fatigue-induced suppression in either group.

**Figure 5:**
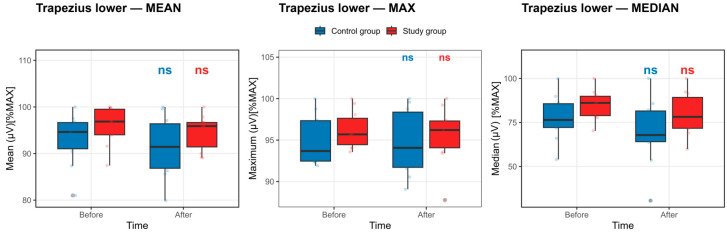
Lower trapezius muscle activation before and after the competition exposure. Statistical significance *p* < 0.05, defined by the Benjamini–Hochberg false discovery rate, else nonsignificant. Boxplots show median and IQR; whiskers = 1.5 × IQR. ns: non-significant.

Trapezius Lower Muscle: As summarized in Supplementary Table S4 and illustrated in Figure 5, no statistically significant within-group pre–post changes in normalized lower trapezius EMG activation were identified in either the control or study group after Benjamini–Hochberg false discovery rate correction (all pFDR ≥ 0.30).

In the control group, maximal EMG amplitude decreased slightly and nonsignificantly from baseline to post-competition exposure (95.00 ± 3.06 vs. 94.70 ± 3.96), with a similarly minor, nonsignificant reduction in mean activation (93.13 ± 5.59 vs. 91.40 ± 6.58). Median activation exhibited a larger numerical decline (77.77 ± 12.99 to 69.64 ± 19.13); however, this change did not reach statistical significance after FDR adjustment. These trends are visually apparent in Figure 5 as a modest downward shift in the central tendency, accompanied by substantial overlap in the interquartile ranges.

In contrast, the study group demonstrated largely preserved lower trapezius activation across all EMG descriptors. Maximal and mean amplitudes showed minimal pre–post differences (96.23 ± 2.25 vs. 95.65 ± 3.48 and 95.92 ± 4.14 vs. 94.70 ± 3.85, respectively), while median values displayed variability without a statistically significant change. Correspondingly, Figure 5 shows closely overlapping pre- and post-competition exposure distributions for all parameters in the study group.

Taken together, the combined evidence from Supplementary Table S4 and Figure 5 suggests relatively greater stability of lower trapezius muscle activation in elite strongmen compared with controls. However, no within-group changes reached statistical significance.

**Figure 6:**
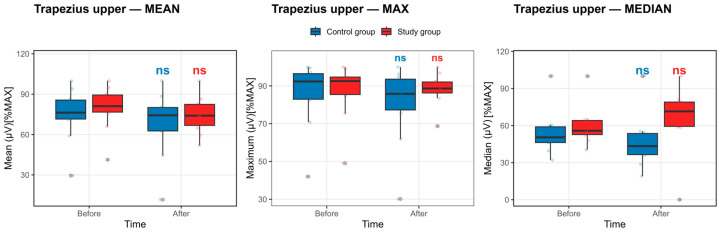
Upper trapezius muscle activation before and after the competition exposure. Statistical significance *p* < 0.05, defined by the Benjamini–Hochberg false discovery rate, else nonsignificant. Boxplots show median and IQR; whiskers = 1.5 × IQR.

Trapezius Upper Muscle: As detailed in Supplementary Table S5 and illustrated in Figure 6, within-group analyses revealed no statistically significant pre–post changes in normalized upper trapezius EMG activation in either the control or study group after Benjamini–Hochberg false discovery rate correction (all pFDR ≥ 0.41).

In the control group, maximal upper trapezius activation showed a small, nonsignificant numerical decrease from baseline to post-competition exposure (85.57 ± 17.78 vs. 80.28 ± 20.80). Similar nonsignificant reductions were observed in mean (74.81 ± 19.83 vs. 67.86 ± 24.96) and median (54.17 ± 18.36 vs. 47.05 ± 21.87) activation. These changes are reflected in Figure 6 as a modest downward trend in central values, accompanied by wide interquartile ranges and substantial overlap between pre- and post-competition exposure distributions, indicating high inter-individual variability.

In the study group, upper trapezius activation remained comparatively stable across all EMG descriptors. Maximal and mean amplitudes demonstrated minimal pre–post differences (87.01 ± 15.14 vs. 88.31 ± 8.49 and 79.73 ± 16.67 vs. 74.77 ± 13.88, respectively), while median values showed variability without a consistent directional change. Correspondingly, Figure 6 illustrates closely overlapping boxplots for baseline and post-competition exposure measurements in the study group.

For the upper trapezius, the control group showed numerically lower post-competition exposure values across MAX, MEAN, and MEDIAN descriptors, although none of these changes reached statistical significance after FDR correction. These reductions are visualized in Figure 6 and quantified in Supplementary Table S5. In contrast, the study group showed stable or modestly increased activation across three outcome parameters.

**Figure 7:**
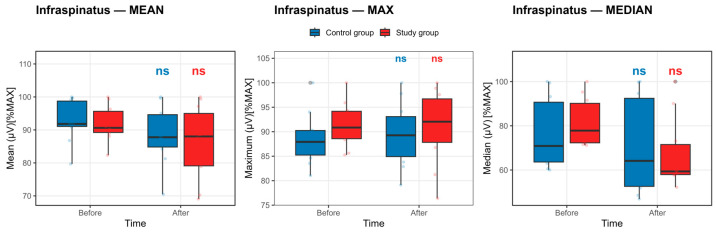
Infraspinatus muscle activation before and after the competition exposure. Statistical significance *p* < 0.05, defined by the Benjamini–Hochberg false discovery rate, else nonsignificant. Boxplots show median and IQR; whiskers = 1.5 × IQR. ns: non-significant.

Infraspinatus Muscle: As reported in Supplementary Table S6 and illustrated in Figure 7, within-group analyses demonstrated no statistically significant pre–post changes in normalized infraspinatus EMG activation in either the control or study group after Benjamini–Hochberg false discovery rate correction (all pFDR ≥ 0.41).

In the control group, maximal infraspinatus activation remained essentially unchanged from baseline to post-competition exposure (88.66 ± 5.45 vs. 89.45 ± 6.56). The mean and median EMG amplitudes showed numerically lower post-competition exposure values (mean: 92.90 ± 6.48 to 88.61 ± 8.95; median: 76.65 ± 16.11 to 71.04 ± 21.95); however, these differences did not reach statistical significance after FDR adjustment. These trends are reflected in Figure 7 as a slight downward shift in the central tendency of the mean and median, accompanied by broad interquartile ranges and substantial overlap across time points.

In the study group, infraspinatus activation exhibited a similar pattern of stability. Maximal, mean, and median EMG amplitudes demonstrated modest numerical decreases (MAX: 91.41 ± 4.66 to 91.00 ± 7.65; MEAN: 91.99 ± 5.43 to 85.90 ± 11.25; MEDIAN: 81.61 ± 10.83 to 67.48 ± 15.77), yet none of these changes were statistically significant after correction. Consistent with these findings, Figure 7 shows largely overlapping pre- and post-competition exposure distributions across all EMG descriptors.

Overall, the combined evidence from Supplementary Table S6 and Figure 7 indicates no statistically significant change in infraspinatus muscle activation following the competition protocol in both groups, with observed numerical changes reflecting inter-individual variability rather than a systematic fatigue-related decline.

**Figure 8:**
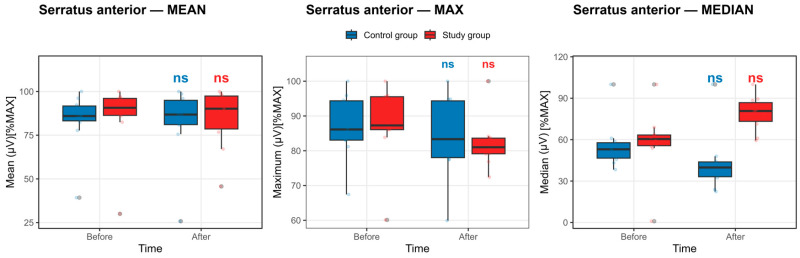
Serratus anterior muscle activation before and after the competition exposure. Statistical significance *p* < 0.05, defined by the Benjamini–Hochberg false discovery rate, else nonsignificant. Boxplots show median and IQR; whiskers = 1.5 × IQR. ns: non-significant.

Serratus anterior muscle: As summarized in Supplementary Table S7 and illustrated in Figure 8, within-group analyses revealed no statistically significant pre–post changes in normalized serratus anterior EMG activation in either the control or study group after Benjamini–Hochberg false discovery rate correction (all pFDR ≥ 0.58).

In the control group, maximal and mean serratus anterior activation showed small, nonsignificant numerical decreases from baseline to post -competition exposure (MAX: 87.14 ± 9.43 to 84.36 ± 11.83; MEAN: 83.51 ± 16.85 to 82.40 ± 21.45). Median activation demonstrated a larger numerical decline (55.72 ± 17.06 to 42.84 ± 21.78); however, this change did not reach statistical significance after FDR adjustment. These patterns are reflected in Figure 8 as a downward trend in median values, with wide interquartile ranges and substantial overlap between pre- and post-competition exposure distributions.

In contrast, the study group exhibited stable serratus anterior activation across EMG descriptors. Maximal and mean amplitudes showed minimal pre–post differences (MAX: 87.76 ± 11.16 to 82.10 ± 7.23; MEAN: 85.60 ± 20.35 to 84.68 ± 17.30), while median activation displayed a numerical increase (58.31 ± 24.12 to 79.17 ± 12.56) that did not achieve statistical significance following correction. Consistent with these findings, Figure 8 demonstrates closely overlapping distributions for baseline and post-competition exposure measurements in the study group.

Collectively, the concordant evidence from Supplementary Table S7 and Figure 8 indicates that serratus anterior neuromuscular activation is preserved following the competition protocol in both groups. Observed numerical fluctuations, including the opposing median trends between groups, reflect inter-individual variability rather than a consistent fatigue-related suppression of serratus anterior activity.

The difference-in-differences analysis (Supplementary Table S8) revealed distinct patterns of neuromuscular responses between the study and control groups across examined muscle regions and EMG signal descriptors (MAX, MEAN, MEDIAN). Linear mixed-effects models were successfully fitted for the majority of region–category combinations; however, in selected strata, contrasts were non-estimable due to limited within-cell variability or model convergence constraints, and these cases were retained for transparency but excluded from inferential interpretation.

Across muscle regions, the DiD estimates generally indicated attenuated reductions or no statistically significant change inEMG activity in the study group compared with the control group. This effect was most consistently observed for mean and median EMG amplitude, whereas maximal EMG values demonstrated greater variability and fewer statistically robust contrasts.

After adjustment for multiple testing using the Benjamini–Hochberg false discovery rate procedure, only a limited subset of contrasts remained statistically significant, underscoring the correction’s conservative nature. Where significant, FDR-adjusted effects consistently favored the study group, indicating a more stable neuromuscular activation profile over time relative to controls. No contrasts demonstrated a significantly greater decline in EMG activity in the study group compared with the control group after FDR correction.

The control group showed a broader pattern of negative pre-post changes across several muscle regions, which contributed to positive DiD estimates in favor of the study group. In contrast, the study group showed smaller absolute changes and reduced dispersion, suggesting a greater tolerance to the applied training or competitive stimulus. Importantly, these group differences were detected despite substantial inter-individual variability, as reflected in the standard errors and degrees of freedom that varied across models.

## 4. Discussion

To our knowledge, this is the first study to collect sEMG data during an actual Strongman Champions League competition rather than an artificial laboratory protocol. During this study, we monitored seven key shoulder-girdle muscles—covering the anterior, middle, and posterior deltoid; upper and lower trapezius; serratus anterior; and infraspinatus—thereby providing a multi-muscle perspective on activation strategies and fatigue resistance. The observed physiological adaptations were compared with an age- and activity-matched control group from Tier 2: Trained/Developmental strongmen. This allowed us to disentangle adaptations specific to strongman training from those attributable merely to general physical activity.

The principal finding of the present investigation was that, despite exposure to an intensive competition protocol, normalized EMG amplitude metrics did not demonstrate a uniform or systematic reduction across the examined shoulder and scapular musculature. Within-group analyses showed that, in the study group, maximal, mean, and median EMG amplitudes were largely preserved across the anterior, medial, and posterior deltoid, upper and lower trapezius, serratus anterior, and infraspinatus muscles, with no consistent pattern of fatigue-related decline. In contrast, the control group exhibited more consistent negative pre–post trends across several muscles, particularly for mean and median EMG amplitudes, often accompanied by moderate-to-large standardized effect sizes despite the absence of FDR-corrected statistical significance.

Difference-in-differences analyses further supported this pattern, indicating that changes over time in the study group were generally comparable to, or more favorable than, those observed in the controls. Notably, a significant ΔΔ effect was identified for the median EMG amplitude of the lower trapezius, favoring the study group after FDR correction, suggesting a relative enhancement or no statistically significant change in neuromuscular output in this muscle. For the remaining muscles and EMG descriptors, ΔΔ estimates were directionally heterogeneous but consistently failed to show greater activation loss in the study group. Collectively, these findings indicate that intensive competitive exposure did not induce generalized neuromuscular impairment; rather, muscle activation responses were regulated in a muscle- and metric-specific manner, with particular stability observed in scapular stabilizers. These outcomes align with the extant literature on neuromuscular adaptations in highly trained athletes, which documents enhanced stabilization of muscle activation during consistent fatigue [27].

Furthermore, the findings presented here do not confirm those reported by Ahtiainen et al. [28], who demonstrated that untrained subjects are more susceptible to declines in strength and muscle activation following a period of training intensification. The lack of a statistically significant change in normalized EMG amplitude observed in elite strongman athletes (Tier 4) is consistent with training-associated neuromuscular adaptations that enhance fatigue resistance during extreme loading. However, because the present study relied on conventional sEMG amplitude measures without motor unit decomposition, the specific neural mechanisms underlying these adaptations cannot be directly resolved. In particular, preferential motor unit recruitment, altered discharge behavior, or changes in recruitment order remain plausible but unverified explanations. Accordingly, references to motor unit–level adaptations should be interpreted as biologically informed hypotheses supported by prior work in strength-trained populations, rather than as direct mechanistic conclusions derived from the current data.

Support for this interpretation can be drawn from studies in strength-trained and elite athletic populations reporting distinct EMG fatigue responses compared with untrained individuals during high-load or prolonged tasks [29]. Contemporary fatigue frameworks further emphasize that preserved or altered EMG amplitudes during maximal voluntary contractions reflect the integrated regulation of central motor drive, peripheral contractile capacity, and task-specific neuromuscular strategies rather than isolated motor-unit behavior per se. In this context, fatigue is increasingly viewed as a multidimensional psychophysiological state arising from the dynamic interaction between performance-related factors (e.g., neural activation and contractile function) and centrally mediated regulatory processes aimed at preserving homeostasis under extreme loading conditions [30].

Also, narrative syntheses of central and peripheral fatigue mechanisms indicate that long-term exposure to high-intensity resistance training can attenuate inhibitory afferent feedback, enhance tolerance to metabolite accumulation, and sustain voluntary activation during maximal or near-maximal tasks, thereby producing EMG responses that differ qualitatively from those observed in untrained individuals [31].

Moreover, previous work in powerlifters, weightlifters, and overhead athletes has demonstrated preserved or altered EMG activation patterns under fatigue, suggesting long-term neuromuscular adaptations to extreme loading demands [13,17,19]. Nevertheless, direct confirmation of motor unit–specific strategies requires high-density EMG or decomposition-based analyses, which should be incorporated in future competition-based investigations.

In contrast, untrained individuals lack these integrated adaptations. Their neuromuscular transmission demonstrates greater variability under fatigue, resulting from intracellular acidification due to limited buffering capacity and less economical motor unit recruitment.

By contrasting the findings of Naderza et al. [32], who observed significant changes in muscle activity, particularly in the infraspinatus and the lower trapezius in trained archers, a conclusion can be drawn that the training specificity of the strongman, characterized by exceptionally high loads and intensities, may induce more advanced neuromuscular adaptations, thereby preserving muscle activation even under substantial fatigue.

The present findings have direct implications for the design of training programs for strongman athletes. The preserved normalized EMG activity of shoulder-girdle muscles observed in elite competitors indicates that performance under competition conditions depends not only on maximal strength of prime movers, but also on the ability to maintain neuromuscular control of scapular stabilizers and rotator cuff muscles under extreme fatigue. Consequently, strongman training should incorporate targeted conditioning of the serratus anterior, upper and lower trapezius, and infraspinatus, with particular emphasis on fatigue resistance rather than isolated maximal activation. Such training should be performed under competition-like conditions, including prolonged time-under-tension, isometric or quasi-isometric loading, and sequencing after heavy compound or carrying tasks. These strategies may enhance joint stability, preserve force transmission across successive events, and reduce the risk of fatigue-related shoulder dysfunction. In contrast, numerically lower post-competition reductions in EMG activity were observed in Tier 2: Trained/Developmental-trained individuals, suggesting insufficient stabilization capacity under cumulative load, highlighting the importance of progressive exposure to event-specific fatigue in less experienced athletes.

This study demonstrates that elite strongman athletes preserve normalized shoulder-girdle muscle activation following a competition-like protocol. Tier 2 active controls exhibited consistent negative pre–post trends in neuromuscular activation that did not survive correction for multiple testing across multiple shoulder muscles. Importantly, these adaptations are muscle-specific, reflecting differential fatigue resistance across the shoulder complex.

Specifically, activation of the anterior, medial, and posterior deltoid muscles remained stable in elite strongmen across all EMG outcome measures. In contrast, the control group demonstrated numerically lower post-competition values across several EMG descriptors, reflecting greater susceptibility to fatigue-related variability rather than statistically significant activation loss. The upper trapezius exhibited a unique response pattern, with elite athletes showing numerically higher mean activation values, suggesting an adaptive role in sustained scapular elevation and load transmission during competition. In contrast, control participants demonstrated numerically lower post-competition values across all upper trapezius descriptors, reflecting greater fatigue-related variability rather than statistically confirmed activation loss.

The lower trapezius and infraspinatus, key contributors to scapular stabilization and glenohumeral joint control, showed preserved activation in elite athletes. In contrast, Tier 2 participants exhibited directionally negative but nonsignificant pre–post trends. This finding highlights the potential role of stabilizing musculature in maintaining neuromuscular activation patterns during extreme loading. Finally, the serratus anterior displayed a distinct response pattern in elite strongmen, characterized by numerically higher maximal activation values that did not reach statistical significance after correction, potentially reflecting enhanced scapulothoracic control under high-demand conditions.

Collectively, these muscle-specific findings indicate that elite strongman athletes employ advanced neuromuscular strategies that enable no statistically significant change in both prime-mover and stabilizer activation during competition. These adaptations may contribute to sustained performance capacity and may reduce the risk of fatigue-related shoulder dysfunction under extreme competitive demands.

This study has several limitations. First, the sample included only ten male elite strongmen and ten Tier 2: Trained/Developmental individuals as controls, limiting generalisability and precluding sex-specific analyses. Second, measurements were obtained only immediately before and after competition, preventing assessment of longer-term adaptations or recovery kinetics. Third, maximal voluntary contraction (MVC) force output was not directly quantified; therefore, EMG outcomes reflect neuromuscular activation during MVC trials rather than absolute force-generating capacity. Fourth, although competition tasks were matched for duration, relative loading was not normalized to individual strength levels, potentially leading to different physiological stress levels between groups. Finally, conventional surface EMG was used without motor unit decomposition, precluding mechanistic inference regarding motor unit recruitment or discharge behavior.

## 5. Conclusions

In conclusion, the present study demonstrates that elite strongman athletes, characterized by long-term exposure to competition-specific loading, preserve normalized shoulder-girdle muscle activation during maximal voluntary contraction following a single-day, multi-event strongman competition. In contrast, general resistance-trained but non–strongman-adapted individuals consistently showed negative pre–post trends in neuromuscular activation across multiple shoulder muscles after competition.

These findings indicate that the ability to maintain shoulder-girdle activation under extreme, competition-relevant fatigue is a training-specific adaptation associated with elite strongman preparation, rather than a universal response to heavy resistance exercise. Importantly, the observed effects are limited to the defined comparison between elite strongmen and non-specialized, Tier 2-trained controls and should not be extrapolated to other strength-trained populations or athletic disciplines.

Collectively, the results highlight the role of specialized training in developing neuromuscular fatigue resistance of the shoulder complex under real competition conditions and provide mechanistic insight into how elite strongman athletes demonstrate a greater capacity to preserve shoulder-girdle activation patterns under extreme cumulative loading without loss of shoulder-girdle activation.

Future studies should combine surface electromyography with direct quantification of maximal voluntary force to better dissociate neural activation from contractile performance during competition-induced fatigue. The application of high-density EMG and motor-unit decomposition techniques may clarify the recruitment and discharge strategies underlying fatigue resistance in elite strongman athletes. Additionally, normalizing competition loads to individual strength capacity would allow more precise comparison of relative physiological stress between groups. Longitudinal designs incorporating multiple post-competition time points are warranted to characterize recovery kinetics and delayed neuromuscular fatigue. Finally, integrating perceptual fatigue measures and biomechanical analyses may improve understanding of how central regulation and mechanical loading interact to shape performance under extreme competitive conditions.

## Data Availability

The data can be provided upon a reasonable request to the corresponding author.

## Supplementary Materials

The following supporting information can be downloaded at https://www.mdpi.com/article/10.3390/life16030477/s1, Table S1. Within-group before–after changes in deltoid anterior muscle activation; Table S2. Within-group before–after changes in deltoid medial muscle activation; Table S3. Within-group before–after changes in deltoid posterior muscle activation; Table S4. Within-group before–after changes in lower trapezius muscle activation; Table S5. Within-group before–after changes in upper trapezius muscle activation; Table S6. Within-group before–after changes in infraspinatus muscle activation; Table S7. Within-group before–after changes in serratus anterior muscle activation; Table S8. Difference-in-differences (ΔΔ) contrasts comparing pre- to post-intervention changes between the study and control groups for surface electromyography (sEMG) outcomes across muscle regions and signal descriptors.
