# Supplementary material for: The Effects of an Acute Strongman Competition on Electromyographic Responses of the Shoulder Girdle Complex
Source: Life (Basel). 2026 Mar 16;16(3):477. doi: 10.3390/life16030477 (PMC13028316; doi:10.3390/life16030477)
Supplement: Supplementary file 1 [file life-16-00477-s001.zip › life-4127454-supplementary.pdf]

Table S1. Within-group before–after changes in deltoid anterior muscle activation. This table presents within-group before–after contrasts (After − Before) for deltoid anterior muscle activation in the control and study groups. Outcomes are reported for maximal (MAX), mean (MEAN), and median (MEDIAN) EMG signal characteristics. Values are expressed as mean ± SD at baseline (Before) and post-intervention (After), together with the estimated change (Δ ± SE), associated p-values adjusted for multiple comparisons using the Benjamini–Hochberg false discovery rate (BH-FDR), standardized effect sizes (Cohen's d with 95% confidence intervals), and mixed-effects model fit indices (marginal and conditional R^2).

Table. Deltoid anterior — Before–After changes

Within-group Before–After contrast (After − Before), BH-FDR by outcome family

region	Group	Before (mean±SD)	After (mean±SD)	Δ (After–Before) ± SE	p	p_FDR (BH)	Cohen's d [95% CI]	Model R² (marg; cond)	FDR < 0.05
				**MAX**					
Deltoid anterior	Control group	88.47 ± 12.94	85.93 ± 14.84	-2.54 ± 5.61	0.653	0.884	-1.14 [-1.93, -0.31]	m=0.038; c=0.039	FALSE
Deltoid anterior	Study group	91.12 ± 10.64	84.69 ± 11.29	-6.43 ± 5.61	0.259	0.884	-0.71 [-1.40, 0.00]	m=0.038; c=0.039	FALSE
				**MEAN**					
Deltoid anterior	Control group	84.31 ± 14.94	77.96 ± 17.61	-6.35 ± 6.46	0.332	0.712	-1.55 [-2.47, -0.59]	m=0.048; c=0.072	FALSE
Deltoid anterior	Study group	86.64 ± 12.81	84.62 ± 11.76	-2.02 ± 6.46	0.756	0.882	-0.20 [-0.83, 0.43]	m=0.048; c=0.072	FALSE
				**MEDIAN**					
Deltoid anterior	Control group	80.48 ± 11.19	74.70 ± 13.05	-5.78 ± 4.78	0.234	0.410	-1.83 [-2.84, -0.77]	m=0.118; c=0.211	FALSE
Deltoid anterior	Study group	84.39 ± 8.54	84.92 ± 9.37	0.53 ± 4.78	0.913	0.981	0.06 [-0.56, 0.68]	m=0.118; c=0.211	FALSE

Table S2. Within-group before–after changes in deltoid medial muscle activation. This table summarizes within-group changes in deltoid medial muscle activation from before to after for the control and study groups. Maximal, mean, and median EMG parameters are shown as mean ± SD before and after the intervention, along with estimated contrasts (Δ ± SE), BH-FDR-adjusted p-values, Cohen's d effect sizes with 95% confidence intervals, and mixed-effects model R^2 statistics. .

Table. Deltoid medial — Before–After changes

Within-group Before–After contrast (After − Before), BH-FDR by outcome family

region	Group	Before (mean±SD)	After (mean±SD)	Δ (After−Before) ± SE	p	p_FDR (BH)	Cohen's d [95% CI]	Model R² (marg; cond)	FDR < 0.05
				**MAX**					
Deltoid medial	Control group	90.73 ± 6.53	88.40 ± 7.96	-2.33 ± 4.01	0.564	0.884	-1.13 [-1.92, -0.31]	m=0.042; c=0.051	FALSE
Deltoid medial	Study group	93.31 ± 6.01	89.22 ± 13.39	-4.09 ± 4.01	0.315	0.884	-0.29 [-0.92, 0.35]	m=0.042; c=0.051	FALSE
				**MEAN**					
Deltoid medial	Control group	90.00 ± 9.03	86.52 ± 11.10	-3.48 ± 4.13	0.405	0.712	-1.22 [-2.04, -0.37]	m=0.245; c=0.260	FALSE
Deltoid medial	Study group	92.40 ± 8.33	78.53 ± 8.15	-13.87 ± 4.13	0.002	0.026	-1.00 [-1.75, -0.21]	m=0.245; c=0.260	TRUE
				**MEDIAN**					
Deltoid medial	Control group	77.82 ± 12.71	70.66 ± 16.33	-7.16 ± 5.80	0.225	0.410	-1.65 [-2.61, -0.66]	m=0.120; c=0.149	FALSE
Deltoid medial	Study group	83.74 ± 10.71	74.41 ± 11.41	-9.33 ± 5.80	0.116	0.410	-1.26 [-2.09, -0.40]	m=0.120; c=0.149	FALSE

Table S3. Within-group before–after changes in deltoid posterior muscle activation. This table reports within-group before–after contrasts for deltoid posterior muscle activation in both groups. Maximal, mean, and median EMG outcomes are presented as mean ± SD at baseline and post-intervention, with corresponding Δ ± SE estimates, BH-FDR-corrected p-values, Cohen's d effect sizes (95% CI), and marginal and conditional R^2 values from mixed-effects models.

Table. Deltoid posterior — Before–After changes

Within-group Before–After contrast (After − Before), BH-FDR by outcome family

region	Group	Before (mean±SD)	After (mean±SD)	Δ (After−Before) ± SE	p	p_FDR (BH)	Cohen's d [95% CI]	Model R^2 (marg; cond)	FDR < 0.05
				**MAX**					
Deltoid posterior	Control group	93.55 ± 7.99	92.87 ± 9.75	-0.68 ± 3.34	0.838	0.884	-0.32 [-0.95, 0.33]	m=0.005; c=0.007	FALSE
Deltoid posterior	Study group	93.28 ± 7.06	94.29 ± 3.86	-1.01 ± 3.34	0.765	0.884	0.10 [-0.52, 0.72]	m=0.005; c=0.007	FALSE
				**MEAN**					
Deltoid posterior	Control group	91.56 ± 10.15	87.48 ± 13.48	-4.08 ± 4.62	0.384	0.712	-1.11 [-1.89, -0.29]	m=0.054; c=0.059	FALSE
Deltoid posterior	Study group	93.56 ± 8.41	88.41 ± 8.44	-5.15 ± 4.62	0.272	0.712	-0.49 [-1.13, 0.18]	m=0.054; c=0.059	FALSE
				**MEDIAN**					
Deltoid posterior	Control group	70.72 ± 14.22	61.48 ± 18.46	-9.24 ± 6.31	0.152	0.410	-1.68 [-2.64, -0.68]	m=0.124; c=0.412	FALSE
Deltoid posterior	Study group	77.03 ± 10.99	77.18 ± 11.50	0.15 ± 6.31	0.981	0.981	0.01 [-0.61, 0.63]	m=0.124; c=0.412	FALSE

Table S4. Within-group before–after changes in lower trapezius muscle activation. This table presents within-group before–after changes in lower trapezius muscle activation for the control and study groups. Data are reported for maximal, mean, and median EMG signal characteristics as mean ± SD before and after the intervention, together with estimated contrasts (Δ ± SE), BH-FDR-adjusted p-values, Cohen's d effect sizes (95% CI), and mixed-effects model R^2 indices.

Table. Trapezius lower — Before–After changes

Within-group Before–After contrast (After − Before), BH-FDR by outcome family

region	Group	Before (mean±SD)	After (mean±SD)	Δ (After−Before) ± SE	p	p_FDR (BH)	Cohen's d [95% CI]	Model R² (marg; cond)	FDR < 0.05
**MAX**									
Trapezius lower	Control group	95.00 ± 3.06	94.70 ± 3.96	-0.3 ± 4.50	0.541	0.884	-0.17 [-0.79, 0.46]	m=0.049; c=0.051	FALSE
Trapezius lower	Study group	96.23 ± 2.25	95.65 ± 3.48	-0.58 ± 4.50	0.217	0.884	-0.14 [-0.76, 0.49]	m=0.049; c=0.051	FALSE
**MEAN**									
Trapezius lower	Control group	93.13 ± 5.59	91.40 ± 6.58	-1.73 ± 8.54	0.897	0.914	-0.73 [-1.42, -0.01]	m=0.004; c=0.008	FALSE
Trapezius lower	Study group	95.92 ± 4.14	94.70 ± 3.85	-1.22 ± 8.54	0.914	0.914	-0.17 [-0.79, 0.46]	m=0.004; c=0.008	FALSE
**MEDIAN**									
Trapezius lower	Control group	77.77 ± 12.99	69.64 ± 19.13	-8.13 ± 8.67	0.146	0.410	-1.23 [-2.04, -0.38]	m=0.274; c=0.409	FALSE
Trapezius lower	Study group	84.96 ± 8.54	79.59 ± 12.41	-5.37 ± 8.67	0.021	0.300	-0.46 [-1.11, 0.20]	m=0.274; c=0.409	FALSE

Table S5. Within-group before–after changes in upper trapezius muscle activation. This table summarizes within-group before–after contrasts for upper trapezius muscle activation in control and study groups. Maximal, mean, and median EMG parameters are expressed as mean ± SD at baseline and after the intervention, along with Δ ± SE estimates, BH-FDR-corrected p-values, standardized effect sizes (Cohen's d with 95% CI), and mixed-effects model R^2 values.

Table. Trapezius upper — Before–After changes

Within-group Before–After contrast (After − Before), BH-FDR by outcome family

region	Group	Before (mean±SD)	After (mean±SD)	Δ (After−Before) ± SE	p	p_FDR (BH)	Cohen's d [95% CI]	Model R² (marg; cond)	FDR < 0.05
**MAX**									
Trapezius upper	Control group	85.57 ± 17.78	80.28 ± 20.80	-5.29 ± 2.77	0.775	0.884	-1.44 [-2.33, -0.52]	m=0.032; c=0.063	FALSE
Trapezius upper	Study group	87.01 ± 15.14	88.31 ± 8.49	1.3 ± 2.77	0.884	0.884	0.08 [-0.55, 0.69]	m=0.032; c=0.063	FALSE
**MEAN**									
Trapezius upper	Control group	74.81 ± 19.83	67.86 ± 24.96	-6.95 ± 3.73	0.258	0.712	-1.12 [-1.91, -0.30]	m=0.102; c=0.112	FALSE
Trapezius upper	Study group	79.73 ± 16.67	74.77 ± 13.88	-4.96 ± 3.73	0.111	0.712	-0.42 [-1.06, 0.24]	m=0.102; c=0.112	FALSE
**MEDIAN**									
Trapezius upper	Control group	54.17 ± 18.36	47.05 ± 21.87	-7.12 ± 7.44	0.456	0.580	-1.87 [-2.91, -0.80]	m=0.097; c=0.098	FALSE
Trapezius upper	Study group	59.79 ± 16.04	66.03 ± 26.49	6.24± 7.44	0.065	0.410	0.23 [-0.41, 0.85]	m=0.097; c=0.098	FALSE

Table S6. Within-group before–after changes in infraspinatus muscle activation. This table reports within-group before–after changes in infraspinatus muscle activation for both groups. Maximal, mean, and median EMG outcomes are presented as mean ± SD before and after the intervention, together with estimated contrasts (Δ ± SE), BH-FDR-adjusted p-values, Cohen's d effect sizes with 95% confidence intervals, and marginal and conditional R^2 statistics from mixed-effects models.

Table. Infraspinatus — Before–After changes

Within-group Before–After contrast (After − Before), BH-FDR by outcome family

region	Group	Before (mean±SD)	After (mean±SD)	Δ (After–Before) ± SE	p	p_FDR (BH)	Cohen's d [95% CI]	Model R^2 (marg; cond)	FDR < 0.05
**MAX**									
Infraspinatus	Control group	88.66 ± 5.45	89.45 ± 6.56	0.79 ± 1.45	0.839	0.884	0.35 [-0.30, 0.99]	m=0.032; c=0.061	FALSE
Infraspinatus	Study group	91.41 ± 4.66	91.00 ± 7.65	-0.41 ± 1.45	0.690	0.884	-0.04 [-0.66, 0.58]	m=0.032; c=0.061	FALSE
**MEAN**									
Infraspinatus	Control group	92.90 ± 6.48	88.61 ± 8.95	-4.29 ± 2.31	0.458	0.712	-1.48 [-2.37, -0.54]	m=0.099; c=0.104	FALSE
Infraspinatus	Study group	91.99 ± 5.43	85.90 ± 11.25	-6.09 ± 2.31	0.598	0.761	-0.68 [-1.36, 0.03]	m=0.099; c=0.104	FALSE
**MEDIAN**									
Infraspinatus	Control group	76.65 ± 16.11	71.04 ± 21.95	-5.61 ± 6.17	0.196	0.410	-0.87 [-1.59, -0.12]	m=0.129; c=0.208	FALSE
Infraspinatus	Study group	81.61 ± 10.83	67.48 ± 15.77	-14.13 ± 6.17	0.390	0.580	-1.65 [-2.61, -0.66]	m=0.129; c=0.208	FALSE

Table S7. Within-group before–after changes in serratus anterior muscle activation. This table presents within-group before–after contrasts for serratus anterior muscle activation in the control and study groups. Outcomes are reported for maximal, mean, and median EMG signal characteristics as mean ± SD at baseline and post-intervention, with corresponding Δ ± SE estimates, BH-FDR-corrected p-values, Cohen’s d effect sizes (95% CI), and mixed-effects model R^2 indices.

Table. Serratus anterior — Before–After changes

Within-group Before–After contrast (After − Before), BH-FDR by outcome family

region	Group	Before (mean±SD)	After (mean±SD)	Δ (After−Before) ± SE	p	p_FDR (BH)	Cohen's d [95% CI]	Model R² (marg; cond)	FDR < 0.05
**MAX**									
Serratus anterior	Control group	87.14 ± 9.43	84.36 ± 11.83	-2.78 ± 7.25	0.470	0.884	-0.98 [-1.73, -0.20]	m=0.034; c=0.056	FALSE
Serratus anterior	Study group	87.76 ± 11.16	82.10 ± 7.23	-5.66 ± 7.25	0.858	0.884	-0.44 [-1.08, 0.22]	m=0.034; c=0.056	FALSE
**MEAN**									
Serratus anterior	Control group	83.51 ± 16.85	82.40 ± 21.45	-1.11 ± 8.62	0.426	0.712	-0.21 [-0.83, 0.42]	m=0.046; c=0.069	FALSE
Serratus anterior	Study group	85.60 ± 20.35	84.68 ± 17.30	-0.92 ± 8.62	0.569	0.761	-0.03 [-0.65, 0.59]	m=0.046; c=0.069	FALSE
**MEDIAN**									
Serratus anterior	Control group	55.72 ± 17.06	42.84 ± 21.78	-12.88 ± 9.42	0.455	0.580	-2.26 [-3.45, -1.05]	m=0.094; c=0.171	FALSE
Serratus anterior	Study group	58.31 ± 24.12	79.17 ± 12.56	20.86 ± 9.42	0.512	0.597	0.94 [0.17, 1.68]	m=0.094; c=0.171	FALSE

Table S8. Difference-in-differences (ΔΔ) contrasts comparing pre- to post-intervention changes between the study and control groups for surface electromyography (sEMG) outcomes across muscle regions and signal descriptors. Estimates represent the ΔΔ effect (Study − Control) derived from linear mixed-effects models, with associated standard errors (SE), degrees of freedom (df), t-ratios, and unadjusted p-values. To account for multiple comparisons across regions and outcome categories (MAX, MEAN, MEDIAN), p-values were adjusted using the Benjamini–Hochberg false discovery rate (FDR) procedure (pFDR). The column pFDR_sig indicates statistical significance after FDR correction. Non-estimable contrasts or model convergence issues, where present, are indicated in the corresponding columns.

Difference-in-Differences (ΔΔ) by Region × Category

Category	Contrast	Estimate	SE	df	t	p	p (FDR)	FDR ≤ 0.05	Failed	Error
**Deltoid anterior**										
MAX	ΔΔ (Study - Control)	-3.89	3.54	27	-1.09818318	0.2818	0.7397	FALSE	FALSE	
MEAN	ΔΔ (Study - Control)	4.33	3.95	27	1.09675637	0.2824	0.7397	FALSE	FALSE	
MEDIAN	ΔΔ (Study - Control)	6.31	3.52	27	1.79081938	0.0845	0.4438	FALSE	FALSE	
**Deltoid medial**										
MAX	ΔΔ (Study - Control)	-1.75	4.71	27	-0.37234388	0.7125	0.8882	FALSE	FALSE	
MEAN	ΔΔ (Study - Control)	-10.40	4.80	27	-2.16481787	0.0394	0.4068	FALSE	FALSE	
MEDIAN	ΔΔ (Study - Control)	-2.18	3.09	27	-0.70440317	0.4872	0.8882	FALSE	FALSE	
**Deltoid posterior**										
MAX	ΔΔ (Study - Control)	1.70	3.50	27	0.48497718	0.6316	0.8882	FALSE	FALSE	
MEAN	ΔΔ (Study - Control)	-1.08	4.17	27	-0.25831148	0.7981	0.9076	FALSE	FALSE	

Difference-in-Differences (ΔΔ) by Region × Category

Category	Contrast	Estimate	SE	df	t	p	p (FDR)	FDR ≤ 0.05	Failed	Error
MEDIAN	ΔΔ (Study - Control)	9.38	6.07	27	1.54620016	0.1337	0.5249	FALSE	FALSE	
**Infraspinatus**										
MAX	ΔΔ (Study - Control)	-0.29	1.67	27	-0.17244493	0.8644	0.9076	FALSE	FALSE	
MEAN	ΔΔ (Study - Control)	0.50	2.74	27	0.18403091	0.8554	0.9076	FALSE	FALSE	
MEDIAN	ΔΔ (Study - Control)	2.75	5.41	27	0.50940632	0.6146	0.8882	FALSE	FALSE	
**Serratus anterior**										
MAX	ΔΔ (Study - Control)	6.60	6.47	27	1.01952216	0.3170	0.7397	FALSE	FALSE	
MEAN	ΔΔ (Study - Control)	1.99	5.09	27	0.39083028	0.6990	0.8882	FALSE	FALSE	
MEDIAN	ΔΔ (Study - Control)	13.36	9.02	27	1.48178326	0.1500	0.5249	FALSE	FALSE	
**Trapezius lower**										
MAX	ΔΔ (Study - Control)	-2.88	4.13	27	-0.69812946	0.4911	0.8882	FALSE	FALSE	
MEAN	ΔΔ (Study - Control)	0.19	9.05	27	0.02126387	0.9832	0.9832	FALSE	FALSE	
**MEDIAN**	**ΔΔ (Study - Control)**	**33.76**	**9.13**	**27**	**3.69909688**	**<0.001**	**0.0205**	**TRUE**	**FALSE**	
**Trapezius upper**										
MAX	ΔΔ (Study - Control)	-1.20	3.31	27	-0.36352121	0.7190	0.8882	FALSE	FALSE	

Difference-in-Differences (ΔΔ) by Region × Category

Category	Contrast	Estimate	SE	df	t	p	p (FDR)	FDR ≤ 0.05	Failed	Error
MEAN	ΔΔ (Study - Control)	-1.80	3.19	27	-0.56439684	0.5771	0.8882	FALSE	FALSE	
MEDIAN	ΔΔ (Study - Control)	-8.52	4.31	27	-1.97883517	0.0581	0.4068	FALSE	FALSE	
